# Platelet Activation and Chemokine Release Are Related to Local Neutrophil-Dominant Inflammation During Hyperacute Human Stroke

**DOI:** 10.1007/s12975-021-00938-w

**Published:** 2021-08-28

**Authors:** Alexander M. Kollikowski, Mirko Pham, Alexander G. März, Lena Papp, Bernhard Nieswandt, Guido Stoll, Michael K. Schuhmann

**Affiliations:** 1 Department of Neuroradiology, University Hospital of Würzburg, Josef-Schneider-Straße 11, 97080 Würzburg, Germany; 2 Department of Neurology, University Hospital of Würzburg, Josef-Schneider-Straße 11, 97080 Würzburg, Germany; 3 Institute of Experimental Biomedicine, University Hospital and Rudolf Virchow Center, University of Würzburg, Würzburg, Germany

**Keywords:** CXCL4, PF4, CXCL7, NAP-2, Ischemic stroke, Chemokines

## Abstract

Experimental evidence has emerged that local platelet activation contributes to inflammation and infarct formation in acute ischemic stroke (AIS) which awaits confirmation in human studies. We conducted a prospective observational study on 258 consecutive patients undergoing mechanical thrombectomy (MT) due to large-vessel-occlusion stroke of the anterior circulation (08/2018–05/2020). Intraprocedural microcatheter aspiration of 1 ml of local (occlusion condition) and systemic arterial blood samples (self-control) was performed according to a prespecified protocol. The samples were analyzed for differential leukocyte counts, platelet counts, and plasma levels of the platelet-derived neutrophil-activating chemokine C-X-C-motif ligand (CXCL) 4 (PF-4), the neutrophil attractant CXCL7 (NAP-2), and myeloperoxidase (MPO). The clinical-biological relevance of these variables was corroborated by specific associations with molecular-cellular, structural-radiological, hemodynamic, and clinical-functional parameters. Seventy consecutive patients fulfilling all predefined criteria entered analysis. Mean local CXCL4 (+ 39%: 571 vs 410 ng/ml, *P* = .0095) and CXCL7 (+ 9%: 693 vs 636 ng/ml, *P* = .013) concentrations were higher compared with self-controls. Local platelet counts were lower (− 10%: 347,582 vs 383,284/µl, *P* = .0052), whereas neutrophil counts were elevated (+ 10%: 6022 vs 5485/µl, *P* = 0.0027). Correlation analyses revealed associations between local platelet and neutrophil counts (*r* = 0.27, *P* = .034), and between CXCL7 and MPO (*r* = 0.24, *P* = .048). Local CXCL4 was associated with the angiographic degree of reperfusion following recanalization (*r* =  − 0.2523, *P* = .0479). Functional outcome at discharge correlated with local MPO concentrations (*r* = 0.3832, *P* = .0014) and platelet counts (*r* = 0.288, *P* = .0181). This study provides human evidence of cerebral platelet activation and platelet-neutrophil interactions during AIS and points to the relevance of per-ischemic thrombo-inflammatory mechanisms to impaired reperfusion and worse functional outcome following recanalization.

**Supplementary Information:**

The online version contains supplementary material available at 10.1007/s12975-021-00938-w.

## Introduction

The pathobiology of acute ischemic stroke (AIS) encompasses a plethora of intertwined processes within the ischemic territory such as energy failure, excitotoxicity, anoxic depolarization, apoptosis, and, more recently recognized, neuroinflammation [1, 2]. Evidence in rodents and baboons points to a prominent role of platelets and coagulation factors which accumulate early at sites of cerebral infarction [3, 4], and drive inflammation-related tissue damage [5]. Human evidence supporting the causative “thrombo-inflammatory” interplay between thrombotic and inflammatory mechanisms within the compromised ischemic vascular compartment is not available [1], but would have profound implications regarding the relevance and adequate timing of conceptually sound add-on treatments to use before and/or after recanalization therapy for large-vessel-occlusion stroke [6–8]. A major limitation in stroke research is that human samples are restricted mainly to peripheral venous blood or lumbar cerebrospinal fluid (CSF) that are both obtained far remote from and frequently with time delay to the ischemic event [9]. Recently, we and other investigators established the method of endovascular microcatheter aspiration of cerebral-ischemic blood from within the center of the occluded arterial compartment during human AIS [10, 11]. Using this novel approach which reduces interference by effects other than local [10, 11], it was now possible to quantify the cerebral concentrations of the platelet-derived neutrophil-activating chemokine CXC motif ligand (CXCL) 4 (platelet factor 4, PF-4), and the neutrophil attractant CXCL7 (neutrophil-activating peptide 2, NAP-2). These chemokines represent highly abundant releasates upon platelet activation [12, 13], and are considered both (1) as biomarkers of high platelet activity in the systemic circulation[14] and (2) as crucial regulators of neutrophil function [13, 15]. The clinical-biological relevance of our findings was addressed by analyzing specific associations with leukocellular, structural-radiological, and hemodynamic-functional parameters assessed during ischemia, and short-term functional outcome following recanalization.

## Methods

This prospective single-center cross-sectional study (08/2018–05/2020) was approved by the ethics committee of the University of Würzburg (#135/17) and complies with the Declaration of Helsinki. All patients or their legal representatives provided written informed consent. From August 2018 to May 2020, microcatheter aspiration of cerebral arterial blood samples during acute large-vessel-occlusion stroke of the anterior circulation was attempted in 258 consecutive patients undergoing mechanical thrombectomy (MT). Briefly, 1 ml of citrate–phosphate-dextrose-adenine (CPDA-1)–anticoagulated local blood from within the cerebral-ischemic arterial compartment and systemic control samples from the cervical internal carotid artery (ICA) were obtained according to the protocol published previously by our group [10]. As defined per protocol, the human occlusion condition was precisely matched with the most widely practiced middle cerebral artery occlusion (MCAO) models of acute focal ischemic stroke [16]. Details on the inclusion criteria are provided in the Supplemental Information. Blood sampling was followed by sample processing and analysis including differential leukocyte counts, platelet counts, and plasma preparation [10]. After centrifugation to obtain cell-free samples, plasma was immediately stored at − 20 °C. Plasma levels of CXCL4/7 and myeloperoxidase (MPO) were quantified by commercially available kits according to the manufacturers’ specifications: enzyme-linked immune sorbent assay of CXCL4—Thermo scientific [Cat.Nr.: EHPF4], CXCL7—abcam [Cat.Nr.: ab100613]; flow cytometric quantification of MPO—Biolegend [Cat.Nr.: 740561]. The extension of cerebral infarction prior to recanalization was measured through the Alberta Stroke Program Early CT Score (ASPECTS; CT: Somatom Definition AS; Siemens Healthineers, Erlangen, Germany) [17]. Post-processing algorithms (syngo iFLOW software; Siemens Healthineers, Erlangen, Germany) were applied for parametric color coding of digital subtraction angiography (DSA; angiography system: Siemens Artis Q, Siemens Healthineers, Erlangen, Germany) series (4 frames per second) and for the quantification of collateralization by means of retrograde collateral flow as assessed by collateral transit time (relative time to peak opacification, rTTP) [18]. rTTP was determined by using a region-of-interest (ROI) analysis, where predetermined ROI locations reflected the site of cerebral sampling and the vascular target region typical of receiving retrograde collateral supply under occlusion condition. rTTP was calculated by subtracting the time to peak opacification (TTP) of ROI 1 (circular, placed at the lacerum segment of the internal carotid artery, measuring 10.80 mm^2^) from the TTP of ROI 2 (circular, placed centered at the mid-insular sampling location, measuring 309.60 mm^2^). The angiographic degree of final reperfusion following recanalization therapy was graded by the modified treatment in cerebral infarction scale (mTICI). Functional clinical outcome was assessed by means of the modified Rankin Scale (mRS) at hospital discharge.

The manuscript was prepared according to the STROBE (Strengthening the Reporting of Observational Studies in Epidemiology) statement for observational studies [19].

## Statistical Analysis

Statistical analyses were performed using GraphPad Prism (GraphPad Prism 9.0.1, GraphPad Software, San Diego, CA, USA). Gaussian distribution was tested by the D’Agostino and Pearson omnibus normality test. The Wilcoxon signed-rank test was used for comparison of related samples. Spearman’s rank correlation coefficient was applied to determine principal associations between local cerebral-ischemic and clinical-radiological target variables to establish the relevance to human AIS. Data are given as mean with 95% confidence interval (CI), as median with interquartile range [IQR], or as absolute and relative frequency distribution, unless otherwise specified. All *P*-values reported are 2-sided with *P* < 0.05 being considered statistically significant.

## Results

The entire eligible cohort comprised *n* = 364 consecutive patients who were treated by MT between August 2018 and May 2020. *N* = 39 patients were excluded for posterior circulation occlusion and *n* = 21 patients for bilateral or multifocal occlusion location. *N* = 46 patients did not qualify for inclusion upon invasive angiographic imaging either due to spontaneous recanalization or sub-occlusion with residual antegrade flow before MT. Microcatheter aspiration of ischemic blood samples was attempted in *n* = 258 patients with angiographically proven large-vessel-occlusion of the following target sites: ICA‐T, middle cerebral artery M1, and proximal M2 segment. Aspiration of cerebral blood samples succeeded in *n* = 143 patients (55%). Out of these, *n* = 70 consecutive patients (27%) met all a priori defined sampling, interventional, and laboratory criteria of inclusion, and entered data analyses [10]. The full patient flow without missing cases is given in the Supplementary Information. All clinical, radiologic, interventional, and sampling‐related patient characteristics are summarized in Table 1.

**Table 1:** Main clinical, radiologic, interventional, and sampling‐related patient characteristics

Demographics	*n* = 70
Age, y	78 [67–83]
Male	25 (35)
Medical history	
Hypertension	61 (87)
Diabetes mellitus	14 (20)
Hyperlipidemia	23 (33)
Atrial fibrillation	40 (57)
Smoking history	16 (23)
Baseline medication	
Anti-thrombotic medication	34 (49)
Antihypertensive drugs	58 (83)
Clinical characteristics	
Systolic blood pressure, mmHg	159 [149–180]
Diastolic blood pressure, mmHg	86 [71–96]
Heart rate, min^−1^	80 [70–97]
NIHSS at presentation	15 [10-18]
Unknown time of symptom onset	18 (26)
ASPECTS at presentation	8 [7-9]
Treatment	
Thrombolysis	
IV rt-PA	29 (41)
Intervention	
Onset-to-puncture, min	245 [169–336]
Angiographic occlusion location^a^	
M1	48 (68)
M2	20 (29)
ICA	12 (17)
rTTP, sec	3 [2-4]
Stent-retrieval maneuvers	2 [1-3]
Successful recanalization^b^	61 (87)
Duration of MT procedure, min	69 [50–114]
Onset-to-final-recanalization time, min	323 [247–379]
Sampling	
Onset-to-distal sampling time, min	278 [204–360]
Onset-to-carotid sampling time, min	334 [262–401]
Outcome	
mRS at discharge	3 [1-5]
Death during hospital stay	10 (14)

Data are given as median [interquartile range] for continuous variables, and number (percentage) for categorial variables

*NIHSS*, National Institutes of Health Stroke Scale; *ASPECTS*, Alberta Stroke Program Early CT score; *IV rt-PA*, intravenous recombinant tissue plasminogen activator; *M1/M2*, middle cerebral artery section; *ICA*, internal carotid artery; *rTTP*, relative time to peak opacification; *MT*, mechanical thrombectomy; *mRS*, modified Rankin Scale

^a^Including multiple sites per patient

^b^Defined as mTICI (modified treatment in cerebral infarction) scale 2b or 3

**Fig. 1:**
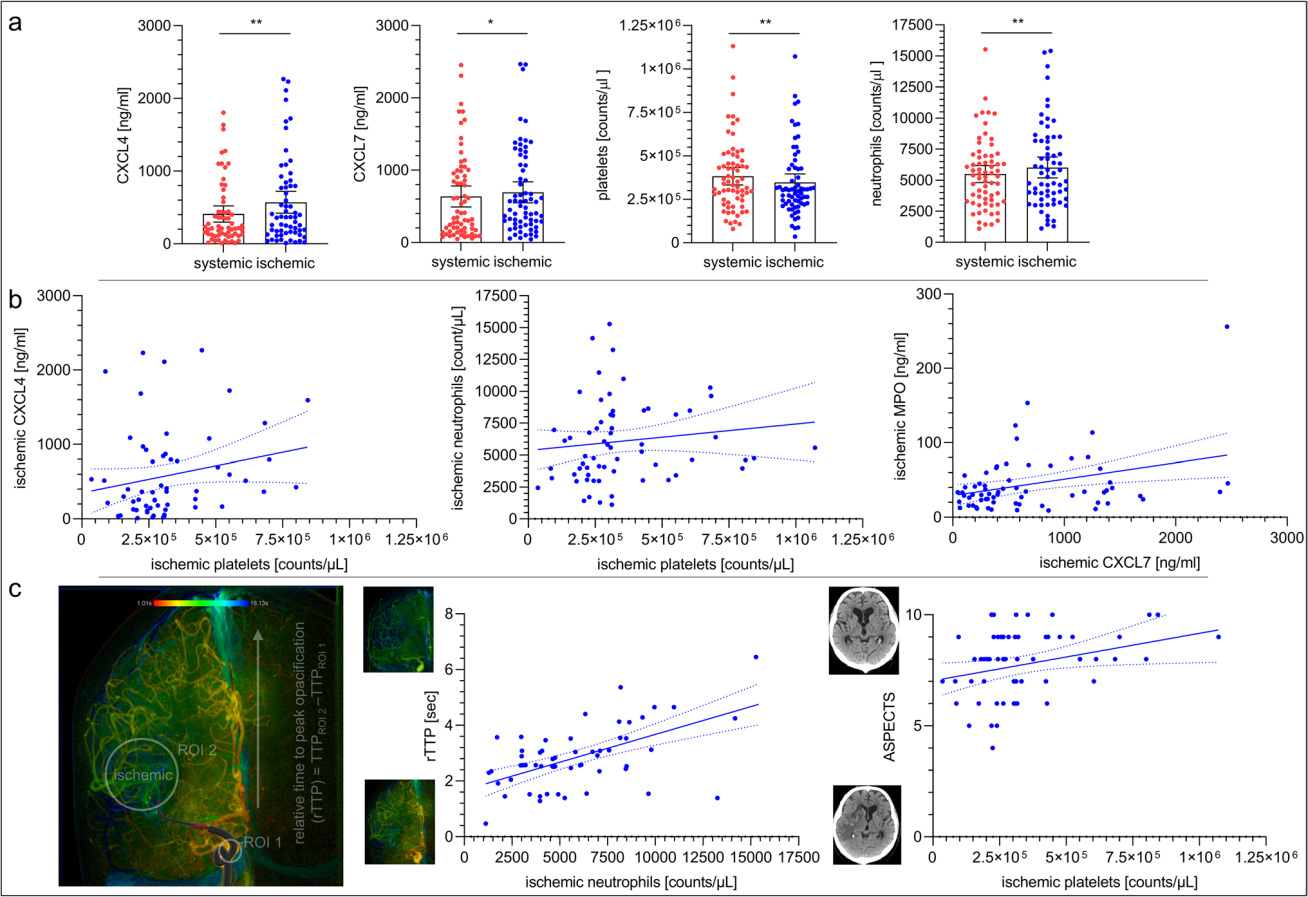
Cerebral platelet activation is paralleled by neutrophil recruitment during acute ischemic stroke. **a** Quantification of plasmatic CXCL4 (PF-4)/CXCL7 (NAP-2) concentrations (*n* = 62/*n* = 67) as well as platelet (*n* = 67) and neutrophil (*n* = 65) counts. Each dot represents related systemic versus cerebral-ischemic blood samples during acute human stroke. Scatter dot plot with mean and 95% confidence interval (CI). Wilcoxon matched-pairs signed-rank test. *P < .05; **P < .005. **b** Left panel: Linear correlation between ischemic CXCL4 concentrations (*y*-axis) and ischemic platelet counts (*x*-axis). Spearman rank order correlation (*n* = 60), *r* = 0.2583, *P* = .0463. Middle panel: Linear correlation between ischemic neutrophil counts (*y*-axis) and ischemic platelet counts (*x*-axis). Spearman rank order correlation (*n* = 64), *r* = 0.2652, *P* = .0342. Right panel: Linear correlation between ischemic MPO (*y*-axis) and CXCL7 (*x*-axis) concentrations. Spearman rank order correlation (*n* = 66), *r* = 0.2446, *P* = .0478. Each dot represents a single case with correlation line. Short-dotted lines: 95% CI of the correlation line. **c** Left panel: Color-coded digital subtraction angiography (DSA) visualizing collateral transit time (relative time to peak opacification, rTTP) and local ischemic blood sampling (target region: ischemic, white circle) during embolic occlusion (red) of the M1 segment of the middle cerebral artery, micro- (distal to embolic lesion), and intermediate catheter (proximal to embolic lesion) in place (black). Middle panel: Linear correlation between collateral transit time (rTTP; *y*-axis, including representative images of good and poor collateral status) and ischemic neutrophil counts (*x*-axis). Spearman rank order correlation (*n* = 57), *r* = 0.4733, *P* = .0002. Right panel: Linear correlation between radiological infarct extension under occlusion (Alberta Stroke Program Early CT score, ASPECTS; *y*-axis, including representative images of progressive and absent infarction) and ischemic platelet counts (*x*-axis). Spearman rank order correlation (*n* = 62), *r* = 0.3075, *P* = .0151. Each dot represents a single case with correlation line Short-dotted lines: 95% CI of the correlation line.

Results of cell and platelet counts, chemokines and clinical correlation analyses are presented in Fig. 1 and the Supplementary Information. Local cerebral-ischemic plasma levels of CXCL4 (+ 39%: 571 ng/ml, 95% CI = 420 to 722 vs 410 ng/ml, 95% CI = 298 to 522, *P* = 0.0095) and CXCL7 (+ 9%: 693 ng/ml, 95% CI = 549 to 838 vs 636 ng/ml, 95% CI = 492 to 781, *P* = 0.013) were higher as compared to intraindividual systemic control samples (Fig. 1a). Concomitantly, cerebral-ischemic platelet counts were reduced by 10% (347,582/µl, 95% CI = 298,711 to 396,453 vs 383,284/µl, 95% CI = 333,194 to 433,374, *P* = 0.0052), whereas neutrophil counts were elevated by + 10% (6022/µl, 95% CI = 5188 to 6855 vs 5485/µl, 95% CI = 4799 to 6171, *P* = 0.0027; Fig. 1a). No significant differences were observed for lymphocyte or monocyte counts, and MPO (Supplementary Information). Subgroup analyses according to whether or not intravenous thrombolysis was performed did not reveal differences with regard to local CXCL4 (656 ng/ml, 95% CI = 401 to 910 vs 506 ng/ml, 95% CI = 316 to 696, *P* = 0.3315), CXCL7 (766 ng/ml, 95% CI = 545 to 988 vs 636 ng/ml, 95% CI = 448 to 828, *P* = 0.2785), and MPO (45 ng/ml, 95% CI = 32 to 57 vs 47 ng/ml, 95% CI = 32 to 62, *P* = 0.8462) concentrations.

Local cerebral-ischemic platelet counts were correlated with CXCL4 (*r* = 0.2583, *P* = 0.0463; Fig. 1b). Neither of the cell counts correlated with CXCL7. Direct correlations were found between local platelet and neutrophil counts (*r* = 0.2652, *P* = 0.0342) as well as local CXCL7 and MPO concentrations (*r* = 0.2446, *P* = 0.0478; Fig. 1b), with MPO being only associated with neutrophil counts (*r* = 0.3257, *P* = 0.0092). Impairment of collateral flow (rTTP) was correlated with the magnitude of cerebral neutrophil influx (*r* = 0.4733, *P* = 0.0002; Fig. 1c). Local platelet counts were related to infarct extension (ASPECTS) prior to recanalization (*r* = 0.3075, *P* = 0.0151; Fig. 1c). CXCL4 concentrations during vascular occlusion were associated with the angiographic degree of reperfusion (mTICI) following recanalization (*r* =  − 0.2523, *P* = 0.0479) and the duration of the MT procedure (*r* = 0.2526, *P* = 0.0495). Functional clinical outcome (mRS) was correlated with local MPO concentrations (*r* = 0.3832, *P* = 0.0014) and platelet counts (*r* = 0.288, *P* = 0.0181). All other results are given in the Supplementary Information.

## Discussion

In this study, we provide evidence for platelet activation and platelet-neutrophil interactions within the collateral circulation of AIS patients as reflected by the local increase of the platelet-derived chemokines CXCL4 and CXCL7 [15]. These observations were accompanied by locally reduced platelet counts and elevated neutrophil counts indicating that platelet activation within the ischemic cerebral vasculature is paralleled by acute local neutrophil-dominant inflammation.

Platelets are the most abundant and most rapidly available source of CXCL4 and CXCL7 which, following platelet activation, are found in thousand-fold higher concentrations than present in normal plasma [15]. While CXCL4 is almost exclusively stored in platelet α-granules [12], CXCL7 is also expressed in peripheral blood mononuclear cells (PBMC), however only in minute amounts [20]. CXCL7 promotes directed intravascular recruitment of neutrophils[21] and induces the degranulation of neutrophil primary and secondary granules [15], the former of which contain large amounts of MPO as one of their major protein constituents [22]. CXCL4, on the other hand, can induce firm adhesion of neutrophils to endothelial cells, while exerting no chemotactic activity [23]. Unlike the plasmatic markers of platelet activation, MPO concentrations did not significantly differ between the sampling sites. This finding is not contradictory to local neutrophil activation, but in line with the interpretation that cerebral ischemia, i.e., a local cerebral reduction in oxygen partial pressure [24], may significantly alter the pattern of neutrophil metabolism [25]. Therefore, the association between CXCL7 and MPO concentrations is highly suggestive for local neutrophil activation and/or modulation by platelets [15]. Lower numbers of platelets, probably caused by attachment to the ischemic endothelium, consumption, and/or platelet necrosis, were associated with more advanced stroke demarcation at presentation [4, 26]. Interestingly, local CXCL4 concentrations were correlated with the angiographic degree of final reperfusion following recanalization therapy and the duration of the intervention, which might reflect both platelet-leukocyte interactions downstream the site of the (macro)vascular embolic lesion and clot interaction with the endothelium at the site of the embolic lesion [23, 27, 28]. Furthermore, the magnitude of cerebral neutrophil influx was associated with impairment of collateral flow [28]. Conceivably, neutrophil influx may lead to collateral failure-associated cerebral infarction, possibly by increasing vascular resistance due to augmented blood viscosity[29] and/or by cellular obstruction [30].

A limitation of the current study is its observational design and use of soluble cell activation markers. In addition, no adjustment for multiple testing was made which can be justified by the exploratory nature of this study. As strength, we provide comparative, direct, and integrative human cerebral data obtained during stroke emergency care which support the pathophysiological concept of stroke-induced “thrombo-inflammation.”[1] Thereby, we shift the experimental focus from thrombo-inflammatory mechanisms during the phase of reperfusion to clinical pertinence already during the earliest phases of stroke formation under occlusive condition [5, 6].

In summary, we report on local cerebral platelet activation and potential platelet-neutrophil interactions within the compromised ischemic cerebral vasculature of large-vessel-occlusion stroke patients and point to the mutual significance of these cells for acute tissue damage. This should prompt further experimental and clinical investigations into local cerebral neutrophil-platelet interactions under occlusion condition that elucidate whether promising anti-thrombotic and/or anti-inflammatory treatments must be administered well before the initiation of recanalization therapies to exert maximum protective effects [8].

## Supplementary Information

Below is the link to the electronic supplementary material.

Supplementary file1 (DOCX 457 KB)

Supplementary file2 (DOC 85 KB)

## Data Availability

The datasets generated during and/or analyzed during the current study are available from the corresponding author on reasonable request.
