# Supplementary material for: Platelet Activation and Chemokine Release Are Related to Local Neutrophil-Dominant Inflammation During Hyperacute Human Stroke
Source: Transl Stroke Res. 2021 Aug 28;13(3):364–9. doi: 10.1007/s12975-021-00938-w (PMC9046342; doi:10.1007/s12975-021-00938-w)
Supplement: Supplementary file 1 — Supplementary file1 (DOCX 457 KB) [file 12975_2021_938_MOESM1_ESM.docx]

**Supplementary Information**

**Platelet activation and chemokine release are related to**

**local neutrophil-dominant inflammation during hyperacute human stroke**

Alexander M. Kollikowski^1^, Mirko Pham^1^, Alexander G. März^1^, Lena Papp^2^,

Bernhard Nieswandt^3^, Guido Stoll^2^, Michael K. Schuhmann^2^

^1^Department of Neuroradiology, University Hospital of Würzburg, Würzburg, Germany;

^2^Department of Neurology, University Hospital of Würzburg, Würzburg, Germany; and

^3^Institute of Experimental Biomedicine, University Hospital and Rudolf Virchow Center, University of Würzburg, Würzburg, Germany

**Supplemental Methods**

Inclusion and exclusion criteria

Patient inclusion criteria were defined as follows: (1) acute ischemic stroke (AIS) with severe neurological baseline deficit qualifying for mechanical thrombectomy (MT) according to current guidelines; (2) multimodal imaging prior to endovascular treatment comprising cranial noncontrast computed tomography (CT), CT-angiography and CT-perfusion scan (complementary) in order (a) to exclude hemorrhage or extensive infarction equivalent to Alberta Stroke Program Early CT Score (ASPECTS) <5, (b) to determine the occlusion site, and (c) to confirm patient eligibility in the extended therapeutic time window ≤24 hours; and (3) periprocedural (invasive angiographic) confirmation of large-vessel-occlusion (LVO) of the following sites: distal internal cerebral artery (ICA-T), middle cerebral artery (MCA) M1 segment, or proximal M2 segment, respectively.

Patients were excluded for the following reasons: (1) proven bilateral or multifocal LVO other than defined; (2) angiographically proven residual or restored antegrade blood flow; (3) any deviation from the interventional, sampling, and preprocessing protocol previously reported by our group;[10] (4) LVO in conjunction with either ≥50% cervical ICA stenosis or ICA dissection; and (5) intraprocedural percutaneous transluminal angioplasty (PTA) or stent implantation.

**Fig. 2** Flowchart of patient inclusion.


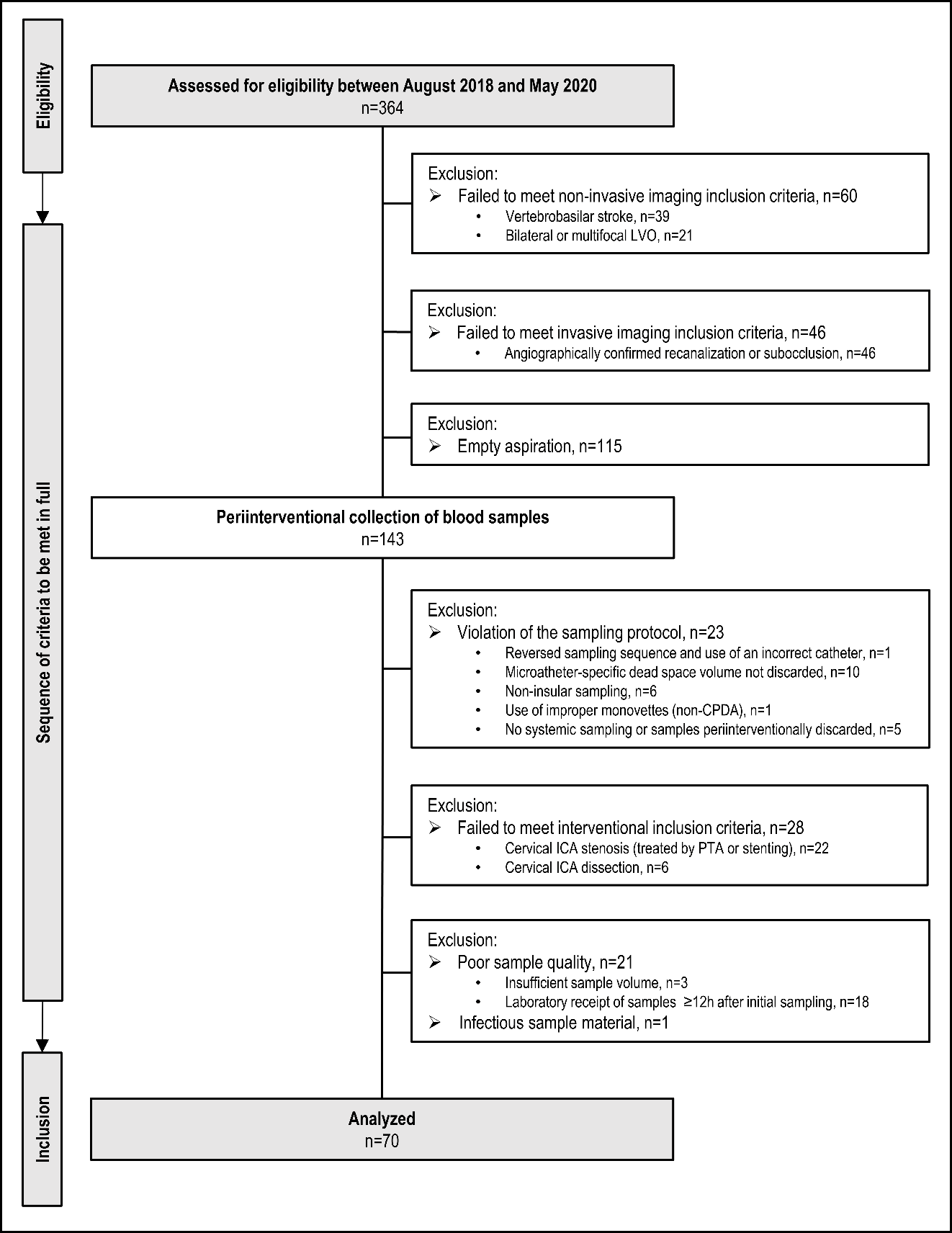


CPDA, citrate–phosphate–dextrose–adenine; ICA, internal carotid artery; LVO, large-vessel-occlusion; PTA, percutaneous transluminal angioplasty.

**Fig. 3** Systemic versus ischemic lymphocyte/monocyte counts and plasmatic MPO concentrations.

**a, b** Systemic versus ischemic lymphocyte (n=65)/monocyte (n=65) counts and **c** plasmatic MPO (n=67) concentrations. Each dot represents related systemic versus cerebral ischemic blood samples during acute human stroke. Scatter dot plot with mean and 95% confidence interval (CI). Wilcoxon matched-pairs signed-rank test. *n.s.*, not significant.

**Table 2** Exploratory correlation analysis between (A) ischemic target variables, (B) pre-interventional stroke severity, (C) collateral transit time, and (D) functional outcome following recanalization therapy.

|  | **y** | **x** | **r** | ***P**** |
| --- | --- | --- | --- | --- |
| **A** | CXCL7, ng/ml | platelets, counts/µL | 0.2221 | .0754 |
|  |  | lymphocytes, counts/µL | 0.2173 | .0846 |
|  |  | monocytes, counts/µL | -0.0704 | .5805 |
|  | neutrophils, counts/µL | CXCL4, ng/ml | 0.0925 | .4860 |
|  |  | CXCL7, ng/ml | 0.1604 | .2054 |
|  | MPO, ng/ml | neutrophils, counts/µL | 0.3257 | .0092 |
|  |  | monocytes, counts/µL | 0.1016 | .4280 |
|  |  | CXCL4, ng/ml | 0.2036 | .1155 |
| **B** | ASPECTS | CXCL4, ng/ml | 0.0499 | .7125 |
|  |  | CXCL7, ng/ml | 0.0073 | .9552 |
|  |  | MPO, ng/ml | -0.2338 | .0674 |
|  |  | neutrophils, ng/ml | -0.2111 | .1055 |
|  |  | lymphocytes, counts/µL | -0.1242 | .3446 |
|  |  | monocytes, counts/µL | -0.1966 | .1321 |
|  | NIHSS | CXCL4, ng/ml | 0.1067 | .4091 |
|  |  | CXCL7, ng/ml | 0.1847 | .1346 |
|  |  | MPO, ng/ml | 0.0485 | .6969 |
|  |  | platelets, counts/µL | -0.1205 | .3314 |
|  |  | neutrophils, counts/µL | 0.1871 | .1355 |
|  |  | lymphocytes, counts/µL | -0.1742 | .1652 |
|  |  | monocytes, counts/µL | 0.1155 | .3598 |
| **C** | rTTP,sec | CXCL4, ng/ml | 0.0626 | .6563 |
|  |  | CXCL7, ng/ml | 0.03812 | .7762 |
|  |  | MPO, ng/ml | 0.0077 | .9543 |
|  |  | platelets, counts/µL | 0.1015 | .4482 |
|  |  | lymphocytes, counts/µL | 0.3132 | .0177 |
|  |  | monocytes, counts/µL | 0.2296 | .0858 |
| **D** | mRS | CXCL4, ng/ml | 0.1711 | .1835 |
|  |  | CXCL7, ng/ml | 0.1623 | .1896 |
|  |  | neutrophils, counts/µL | 0.1626 | .1958 |
|  |  | lymphocytes, counts/µL | -0.0297 | .8145 |
|  |  | monocytes, counts/µL | -0.01346 | .9152 |

ASPECTS, Alberta Stroke Program Early CT Score; NIHSS, National Institutes of Health Stroke Scale; rTTP, relative time to peak opacification; mRS, modified Rankin Scale.

*Statistical analysis: Spearman rank correlation coefficient.

**Table 3** Exploratory correlation analysis of ischemic target variables with (A) the duration of the thrombectomy procedure, (B) number of stent-retrieval maneuvers, and (C) reperfusion status.

|  | **y** | **x** | **r** | ***P**** |
| --- | --- | --- | --- | --- |
| **A** | Duration of MT procedure, min | CXCL7, ng/ml | 0.1614 | .1954 |
|  |  | MPO, ng/ml | 0.1655 | .1841 |
|  |  | platelets, counts/µL | 0.1298 | .2988 |
|  |  | neutrophils, counts/µL | -0.0454 | .7214 |
|  |  | lymphocytes, counts/µL | -0.0637 | .6168 |
|  |  | monocytes, counts/µL | -0.0431 | .735 |
| **B** | Stent-retrieval maneuvers | CXCL4, ng/ml | 0.1534 | .238 |
|  |  | CXCL7, ng/ml | 0.0995 | .4266 |
|  |  | MPO, ng/ml | 0.0802 | .5221 |
|  |  | platelets, counts/µL | 0.1564 | .2099 |
|  |  | neutrophils, counts/µL | -0.0115 | .9283 |
|  |  | lymphocytes, counts/µL | -0.0785 | .5375 |
|  |  | monocytes, counts/µL | -0.0329 | .7966 |
| **C** | mTICI | CXCL7, ng/ml | -0.1672 | .1764 |
|  |  | MPO, ng/ml | -0.0771 | .5351 |
|  |  | platelets, counts/µL | -0.2304 | .0607 |
|  |  | neutrophils, counts/µL | 0.0925 | .4637 |
|  |  | lymphocytes, counts/µL | 0.0074 | .9534 |
|  |  | monocytes, counts/µL | 0.1762 | .1604 |

MT, mechanical thrombectomy; mTICI, modified Treatment in Cerebral Ischemia Scale.

*Statistical analysis: Spearman rank correlation coefficient.
